# Optimization of the autolysis of rainbow trout viscera for amino acid release using response surface methodology

**DOI:** 10.12688/openreseurope.17646.2

**Published:** 2024-11-14

**Authors:** Haizea Domínguez, Bruno Iñarra, Jalel Labidi, Carlos Bald

**Affiliations:** 1 Food Research, AZTI Foundation, Basque Research and Technology Alliance (BRTA), Derio, Bizkaia, 48160, Spain; 2 Biorefinery and Processes Research Group, University of the Basque Country, Donostia-San Sebastian, Gipuzkoa, 20018, Spain

**Keywords:** Fish viscera, autolysis, protein hydrolysates, response surface methodology, free amino acids

## Abstract

**Background:**

Due to the huge amounts of their production in Europe, their environmental impact, and the difficulty in processing them, there is a clear necessity for the valorization of rainbow trout viscera. Considering that the production of fishmeal with viscera can be problematic, and in order to make viscera more profitable, the production of fish protein hydrolysates has been considered. Although silage and enzymatic hydrolysis are the most common methods for obtaining hydrolysates, autolysis has emerged as an alternative method that uses endogenous enzymes of the viscera.

**Methods:**

Considering the stability and characteristics of the enzymes, a factorial design was carried out using three variables: pH, temperature, and water content. The design resulted in 15 experiments, and the results were analyzed using response surface methodology. The optimum parameters were validated by comparing the predicted outcomes with experimental results. Additionally, a kinetics study was conducted to shorten the autolysis time. Results from autolysis were compared with those from silage and enzymatic hydrolysis in a previous study.

**Results:**

The optimal conditions for achieving the highest degree of hydrolysis and yield of free amino acids (FAAs) per 100 g of viscera and per total protein were determined to be a pH of 8, a temperature of 40 °C, and a water content of 6.85%. The pH and content of the added water were found to be significant variables during autolysis ( *p* < 0.05). The kinetic study showed that 7 h was still required to be effective.

**Conclusions:**

Autolysis achieved a lower degree of hydrolysis than silage; however, as it solubilized more protein, the global yield of free amino acids per 100 g of viscera was slightly higher. It was concluded that endogenous alkaline proteases could be used in an autolytic process to obtain a free amino acid-rich hydrolysate from trout viscera.

## Introduction

In recent decades, Spain has witnessed a remarkable expansion in aquaculture, becoming one of the countries with the highest aquaculture production in the European Union, where mussel, seabass, seabream, and rainbow trout are the most produced species. In 2020, almost 123 million tons of fish were produced from aquaculture worldwide, and this number is continuously growing ( FAO, 2022). The increase in fish production has led to a corresponding increase in fish byproducts, posing potential environmental risks ( Marti-Quijal *et al.*, 2020). Among these by-products, which are 60–70% of the total fish weight, heads, skin, scales, bones, viscera, and muscle can be found mainly ( Moreira *et al.*, 2022). It is estimated that nowadays 60% of fish is processed by the transformation industry, which is the main producer of fish by-products ( Arvanitoyannis & Kassaveti, 2008; FAO, 2022).

Rainbow trout is the second most important aquaculture species in Spain, with a production of 16,328 tons by 2022 ( APROMAR, 2023). Within the European Union (EU 27), rainbow trout occupies the first position, with 193,266 tons in 2021. The global aquaculture production of rainbow trout has reached 948,663 tons in 2021 ( APROMAR, 2023), which translates into between 569,198 and 664,064 tonnes of rainbow trout by-products.

Fish viscera constitute the 10–18% of the whole fish weight and belong to the group of by-products used to produce fishmeal. However, its processing in the fishmeal plants presents challenges, including rapid degradation due to high microbial counts in the gastrointestinal tract ( Olsen & Toppe, 2017) and its water content (up to 40%), resulting in significantly high energy costs during the drying process. Despite these hurdles, fish viscera hold promise as a valuable raw material for various applications beyond fishmeal production, including agro-industrial applications or for the extraction of protein hydrolysates ( Villamil *et al.*, 2017).

Fish protein hydrolysates (FPHs) can be obtained through protein hydrolysis to produce free amino acids (FAAs) and low-molecular-weight peptides. Hydrolysis can be acidic, alkaline, or enzymatic, with the latter being the easiest controlled method and the most commonly used one ( Villamil *et al.*, 2017). However, the cost of the enzymes presents an economic challenge, making the process viable only when the final product can reach higher prices in the market. FPHs can be used in many sectors such as animal feed ( COPALIS, 2024), nutraceuticals ( Nirmal *et al.*, 2022), biofertilizers, and biostimulants ( Villamil *et al.*, 2017). A feasible option to valorize fish by-products is to produce FPHs as intermediary products to produce amino acid-based biostimulants, which are substances applied to plants and enhance their nutritional efficiency and abiotic stress tolerance ( du Jardin, 2015).

The use of fish proteases can reduce the economic cost of the process because the price of commercial enzymes is high. As reported by Vannabun *et al.* (2014), fish viscera contain different types of proteases that trigger the breakdown of proteins: serine, aspartyl/acid, cysteine/thiol, and metalloproteases. Trypsin was found inside the serine-type. It is stable and active in the pH range of 7.5 and 8.5 and is found in the pyloric ceca ( Martin, 1994). Trypsin, in addition to cleaving ingested proteins, activates the precursor forms of several other digestive proteases, such as chymotrypsin ( Morrissey & Okada, 2007). In fact, the use of trypsin is increasing because of its unique features: stability and activity over a wide range of pH (8–11) and temperature (38–70 °C) ( Coppola *et al.*, 2021). Meanwhile, pepsin can be found in the aspartyl/acid group, which is located within the fish stomach, and its peak activity is under acidic conditions ( Martin, 1994; Morrissey & Okada, 2007).

Fish silage or acid autolysis is normally used in areas with high fishery rates, but fishmeal processing plants are not economically feasible for obtaining FPHs ( Olsen & Toppe, 2017). It consists of liquefaction and stabilization of minced fish at room temperature ( Olsen *et al.*, 2014). Hydrolysis of proteins occurs due to endogenous acid proteases located at the fish viscera, which trigger the breakdown of proteins into small peptides and free amino acids ( Prabha *et al.*, 2013). However, silage can require several days to achieve a high degree of hydrolysis. Nikoo *et al.* (2021) produced FPHs carrying autolysis of rainbow trout by-products in neutral to slightly alkaline conditions in 1 up to 3 h, where the resulting dominant peptide fractions were dipeptides and tripeptides (51.6-58.4%) and free amino acids (27.3-32.9%).

Response surface methodology (RSM) has been successfully applied to optimize the hydrolysis of various by-products, such as shrimp heads ( Cao *et al.*, 2008), trout viscera ( Taheri *et al.*, 2013), seabream and seabass by-products ( Valcarcel *et al.*, 2020), tuna viscera ( Garofalo *et al.*, 2023) and catla viscera ( Bhaskar *et al.*, 2008).

In a previous study, the comparison between acid autolysis and hydrolysis employing commercial enzymes revealed the remarkable effectiveness of endogenous acid proteases in solubilizing proteins, which was attributed to their significant endoprotease activity. However, these proteases were less efficient in releasing amino acids because of their low exoprotease activity ( Domínguez *et al.*, 2024). Consequently, current research focuses on optimizing the autolytic process using endogenous proteases of rainbow trout viscera. The objective is to maximize the yield of free amino acids while accelerating the process compared to conventional silage or acid autolysis methods, thereby reducing the costs derived from using commercial enzymes, reducing the energy for heating and the use of water.

## Methods

### Fish material processing

All fish viscera used in this study were obtained from cultured rainbow trout ( *Oncorhynchus mykiss*) kindly provided by Caviar Pirinea SL (Yesa, Navarra). The viscera were composed of the stomach, gut, swim bladder, pancreas, kidney, and gonads, without the liver, which is lost during gutting due to its fragility. They were transported in plastic bags on ice in isotherm containers as soon as the fish were eviscerated and stored at –20 °C at their arrival until required for experimentation.

Prior to each process, the viscera were minced using a Grindomix GM 300 knife mill (Retsch GmbH, Haan, Germany), and the resulting mince underwent defatting through decantation at room temperature to avoid the inactivation of native enzymes ( Opheim *et al.*, 2015). The compositions of both the raw material and defatted material were determined ( Table 1).

**Table 1.** Composition of the fresh and defatted viscera.

	Dry matter (%)	Protein (%) d.m.	Ash (%) d.m.
**Fresh viscera**	56.2 ± 4.3	9.4 ± 0.2	1.1 ± 0.03
**Defatted viscera**	48.7 ± 1.9	16.1 ± 0.4	1.4 ± 0.04

### Production of fish viscera hydrolysates

Lab-scale autolysis was carried out in 500 mL batches using Sell Symphony 7100 Bathless Dissolution equipment (Distek Inc., North Brunswick, NJ, USA). To adjust the pH, solutions of 10 M sodium hydroxide (catalogue number 10187850 Fisher Scientific S.L., Madrid, Spain) and 6 M hydrochloric acid (catalogue number 15242380, Fisher Scientific S.L., Madrid, Spain) were used, and measurements were performed using a pH meter (GLP 21+, Crison Instruments, Barcelona, Spain). For hydrolysis processes requiring the addition of water, distilled water was utilized with the appropriate percentage, as detailed in the Results section (Optimization of autolysis conditions).

Following the autolysis process, the reactor contents were heated at 90 °C for 15 min to inactivate the enzymes. Subsequently, centrifugation was performed at 4347 × *g* for 20 minutes in 500 mL containers in an oscillating rotor to separate the solubilized protein from the remaining oil, emulsion interphase, and non-digested solids.

### Chemical analyses

The proximate composition of the samples was analyzed according to the official methods established by the Association of Official Analytical Chemists (AOAC). The dry matter content was determined by drying the samples at 100 °C until they reached a constant weight (method 934.01). The crude protein content was determined using the Kjeldahl method (955.04). The ash content was determined by heating the samples at 500 °C for 24 h and then at 700 °C for 2 h.

The free amino acid content was determined using high-performance liquid chromatography with diode array detection (RP-HPLC-DAD). The samples were prepared by adding 8 mL of HCl 0.1 N to 1 g of sample, followed by agitation for 30 min and filtration through 0.45 µm filters. The samples and standards were derivatized in the injection needle using borax, OPA, and FMOC. The column used in the analysis was Poroshell HPH-C18, 4.6x100 mm, 2.7 µm (Agilent Technologies, Madrid, Spain). The Chromatography was performed using an isocratic system with acetonitrile (45%), methanol (45%), and water (10%). The detection wavelengths were 262 and 338 nm, respectively.

Size exclusion high-performance chromatography (SEC-HPLC) was performed to determine the peptide molecular weight distribution of the hydrolysates. Analytes were separated using an AdvanceBio SEC LC column (300 Å, 7.5 × 300 mm, 2.7 µm (Agilent Technologies, Spain) connected to a diode array detector. Samples were eluted with 50% acetonitrile and 50% water with 0.1% of trifluoroacetic acid as mobile phase at a flow rate of 0.7 mL/min after a 1 µL injection at a temperature of 30 °C. The DAD signal was measured at a wavelength of 214 nm. Each sample was filtered through a 0.45 µm PVDF filter and diluted to a protein concentration of 2 g/L before injection.

### Experimental design

Optimization experiments of the autolysis conditions were conducted by employing response surface methodology (RSM) with a Box-Behnken experimental design. A factorial design was generated considering three factors (content of added water, pH, and temperature) and three levels using the Statgraphics Centurion XVI software (16.2.04 version, Statgraphics Technologies Inc., Virginia, USA). The design shown in Table 2 resulted in 15 experiments, including triplicate measurements of the central point. pH and temperature were selected based on previously published values ( Vannabun *et al.*, 2014) and preliminary experiments, where pH 3 resulted in low yields of free amino acids and experiments at pH 9 produced a mucous emulsion, and agitation of the sample was not feasible. The amount of added water, calculated as the percentage of water in the total reactor volume, was included as a variable to reduce the quantity of water used; in enzymatic hydrolysis and autolysis, the samples are typically diluted 1:1 ( Nikoo *et al.*, 2021; Valcarcel *et al.*, 2020; Vázquez *et al.*, 2019).

**Table 2.** Experimental conditions established to optimize the autolysis.

Factors	Levels		
	-1	0	+1
**Content of added water (%)**	0	25	50
**pH**	6	7	8
**Temperature (°C)**	40	50	60

### Statistical analysis

Statgraphics Centurion XVI software (16.2.04 version, Statgraphics Technologies Inc., Virginia, USA) was used to perform the experimental design and statistical analysis. RSM ANOVA (analysis of variance) was used to determine significant terms included in the model (at *p* < 0.05) while having a Lack of Fit *p*-value higher than 0.05. For the validation of the optimum conditions, results were analyzed with ANOVA and Student’s t-test, and factors were considered significant with a *p*-value of less than 0.05.

## Results and discussion

### Optimization of autolysis conditions

As mentioned, the influence of three different factors (pH, temperature, and content of added water) was determined using a factorial design. The chosen responses were: degree of hydrolysis, yield of FAA from viscera, and yield of FAAs from total protein, and their calculations are explained in Equation (1), Equation (2) and Equation (3). The observed values for each experimental condition are shown in Table 3.

$$\;\text{Degree}\;\text{of}\;\text{hydrolysis}\;(\%)=\frac{\text{Percentage}\;\text{of}\;\text{FAAs}\;\text{in}\;\text{solubilised}\;\text{phase}}{\text{Percentage}\;\text{of}\;\text{protein}\;\text{in}\;\text{solubilised}\;\text{phase}\;*100}\;(1)$$

$$\begin{array}{l} \text{Yield}\;\text{of}\;\text{FAAs}\\ \text{from}\;\text{viscera}\;(\%) \end{array}=\frac{\text{Weight}\;\text{of}\;\mathrm{solubilised}\;\text{phase}\;*\;\text{Percentage}\;\text{of}\;\text{FAAs}\;\text{in}\;\text{solubilised}\;\text{phase}}{\text{Weight}\;\text{of}\;\text{viscera}\;\text{sample}}*100\;(2)$$

$$\;\text{Yield}\;\text{of}\;\text{FAAs}\;\text{from}\;\text{total}\;\text{protein}\;(\%)=\frac{\text{Weight}\;\text{of}\;\text{FAAs}\;\text{in}\;\text{solubilised}\;\text{phase}}{\Sigma \;\text{Weights}\;\text{of}\;\text{the}\;\text{protein}\;\text{in}\;\text{all}\;\text{the}\;\text{phase}}*100\;(3)$$

**Table 3.** Levels of independent variables and the observed values of the responses.

Run number	pH	Temperature (°C)	Content of added water (%)	Degree of hydrolysis (%)	Yield of FAAs from viscera (%)	Yield of FAAs from total protein (%)
1	7	50	25	66.7 ± 0.5	4.0 ± 0.1	54.5 ± 1.2
2	7	40	50	64.9 ± 5.9	4.2 ± 0.1	54.9 ± 0.1
3	8	40	25	69.5 ± 0.1	4.3 ± 0.1	55.5 ± 0.1
4	6	60	25	64.0 ± 1.7	4.1 ± 0.1	53.4 ± 1.5
5	8	60	25	57.3 ± 0.5	4.2 ± 0.1	56.1 ± 0.8
6	6	50	0	62.4 ± 0.2	1.1 ± 0.01	15.0 ± 0.1
7	6	40	25	61.5 ± 0.2	3.7 ± 0.02	49.1 ± 0.3
8	7	60	50	68.7 ± 0.6	4.2 ± 0.1	58.6 ± 0.7
9	7	50	25	68.8 ± 0.9	3.8 ± 0.1	52.1 ± 0.8
10	7	60	0	62.3 ± 1.1	2.9 ± 0.1	42.6 ± 0.8
11	8	50	0	64.1 ± 1.3	5.0 ± 0.1	61.6 ± 1.6
12	6	50	50	67.5 ± 1.8	4.3 ± 0.1	53.7 ± 1.6
13	8	50	50	61.2 ± 1.0	5.4 ± 0.2	60.8 ± 1.8
14	7	40	0	65.5 ± 0.3	3.6 ± 0.02	47.8 ± 0.3
15	7	50	25	72.5 ± 2.2	3.9 ± 0.1	54.7 ± 1.8

**Figure 1.**
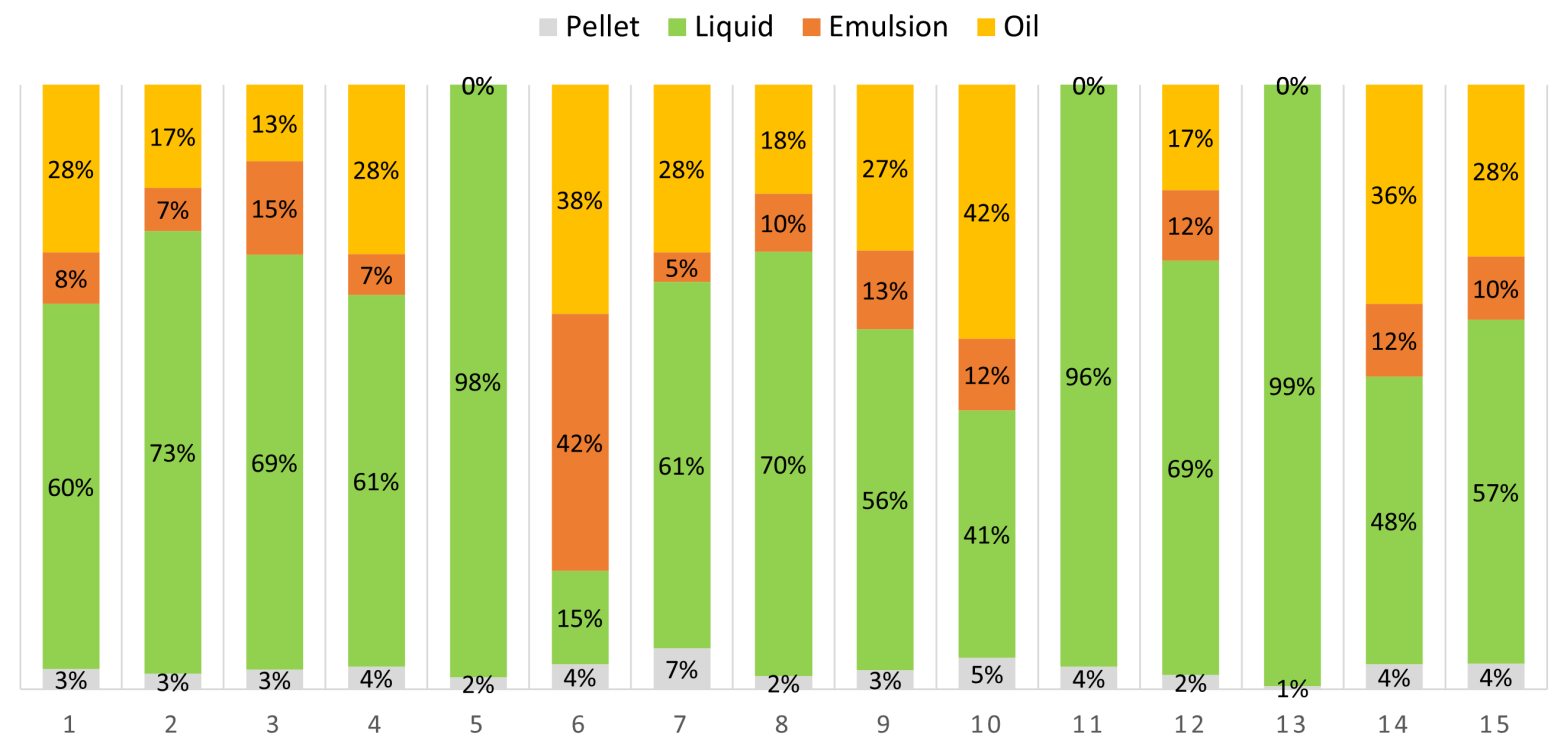
Phase distribution of the hydrolysates of each run of the experimental design.

The composition of the hydrolysates, as illustrated in Figure 1, varied primarily because of the differences in the water content of the samples. In some cases, the hydrolysate was formed by a mucous emulsion with a small pellet (numbers 5, 11, and 13). This might be due to the alkaline pH at high temperatures (50 °C and 60 °C), regardless of the amount of water added, which provoked structural changes in fish proteins that affected their solubility and stability.

The pH (0.024) and the combination of pH and water content (0.047) were significant factors in obtaining the highest degree of hydrolysis and yield of free amino acids from total protein, respectively. However, no significant factors were found for the yield of FAAs from viscera.

**Figure 2.**
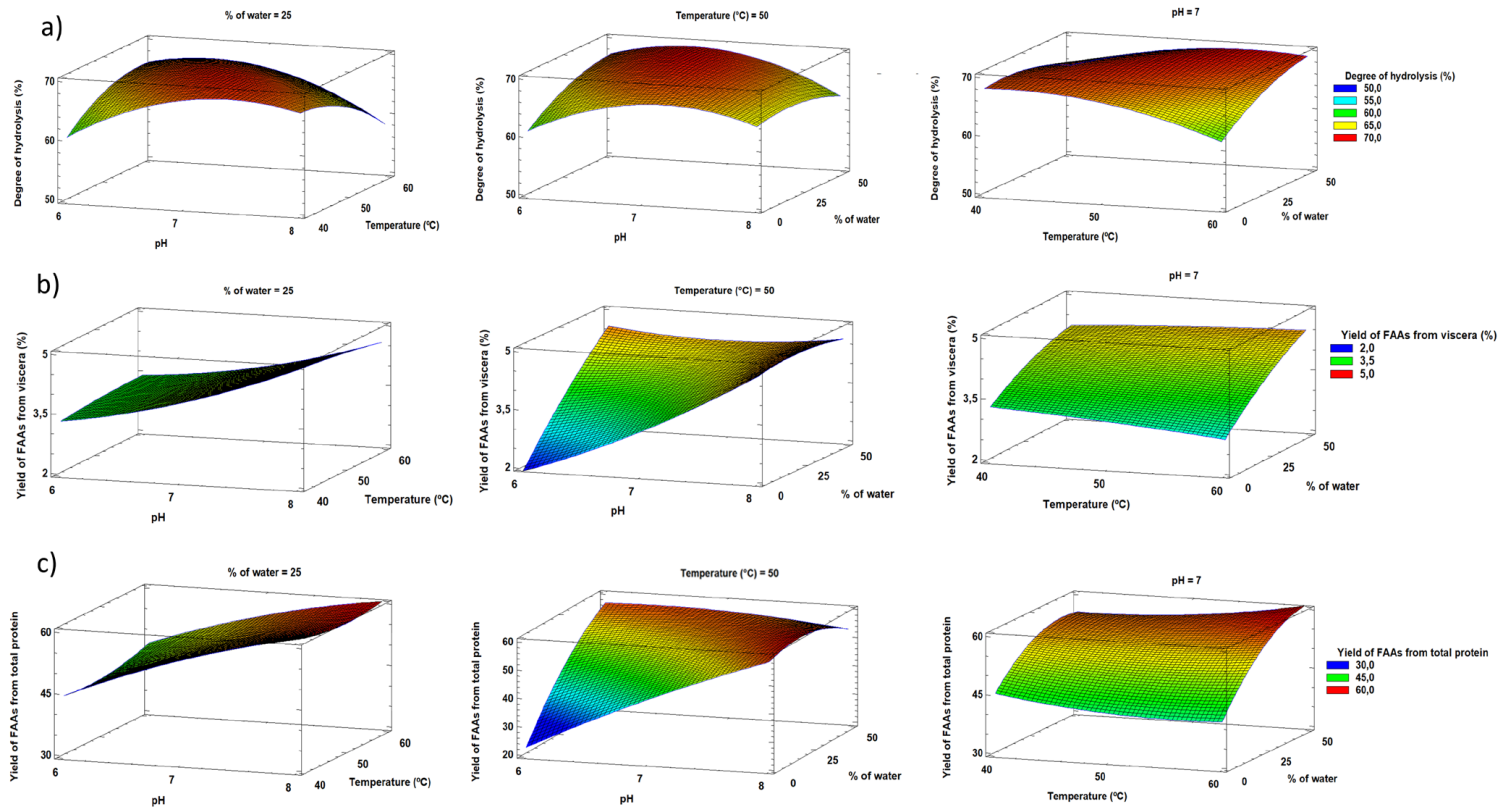
Response surface plots for **a**) degree of hydrolysis, **b**) yield of FAAs from viscera, and **c**) yield of FAAs from total protein.

Figure 2 shows the response surface graphics obtained from Statgraphics. The optimum conditions according to the RSM were pH 8, a temperature of 40 °C, and a 6.85% water content in the sample, with the following predicted results: 68.8% degree of hydrolysis, yield of 5.0% FAAs from viscera, and yield of 62.8% FAAs from total protein. The optimum autolysis conditions obtained were consistent with those reported by Nikoo *et al.* (2021), who obtained the highest total protease activity at 40 °C and pH 8 and described that higher autolysis temperatures could lead to strong oxidizing conditions and amino acid loss. Andevari *et al.* (2019) reported that the pyloric caecal enzyme extract of rainbow trout is very stable at pH 8 and retains more than the 85% of its activity after one hour of heating at 40 °C.

The equations of the full model are shown in Equation (4), Equation (5), and Equation (6), where P corresponds to the pH, T is the temperature, and W is the content of added water. A corresponds to the predicted degree of hydrolysis, B is the yield of FAAs from viscera, and C is the yield of FAAs from total protein. The goodness of fit of the model was checked using the coefficient of determination (R 2). This indicates that the model explained the variability in the degree of hydrolysis by 79.7%, the yield of free amino acids from viscera by 78.4%, and the yield of free amino acids from total protein by 83.7%. To check if the data fit well in the model, the p value of the Lack of Fit was calculated, being for the degree of hydrolysis 0.4603, for the yield of free amino acids from viscera 0.0046, and for the yield of FAAs from total protein 0.022. This suggests that there was only a good fit of the model for the degree of hydrolysis, but neither a good fit for the other responses nor a very good agreement between the experimental and predicted values from the model, probably due to the complexity of the sample and the difficulty of homogenous sample preparation. In fact, other authors like Garofalo *et al*. (2023), obtained an explained variance of 71.58% in their model for the yield of oil extraction in tuna viscera, and van’t Land *et al.* (2017) reported that inhomogeneous sampling could have affected the results for fish silages.

$$\begin{array}{l} \text{A}=-313.668+78.6225*\text{P}+4.3198*\text{T}+0.188737*\text{W}-4.18983*{\text{P}}^{2}\\ \;-0.367423*\text{P}*\text{T}-0.0807647*\text{P}*\text{W}-0.020835*{\text{T}}^{2}\\ \;+0.0107677*\text{T}*\text{W}-0.00222269*{\text{W}}^{2} \end{array}\;(4)$$

$$\begin{array}{l} \text{B}=-0.621275-0.871207*\text{P}+0.0963811*\text{T}+0.198324*\text{W}+0.20565*{\text{P}}^{2}\\ \;-0.0120604*\text{P}*\text{T}-0.0277759*\text{P}*\text{W}-0.000344559*{\text{T}}^{2}\\ \;+0.000685222*\text{T}*\text{W}-0.000230166*{\text{W}}^{2} \end{array}\;(5)$$

$$\begin{array}{l} \text{C}=-151.797+46.3671*\text{P}-1.04083*\text{T}+2.96205*\text{W}-1.72577*{\text{P}}^{2}\\ \;-0.0901883*\text{P}*\text{T}-0.394674*\text{P}*\text{W}+0.014904*{\text{T}}^{2}\\ \;+0.00892304*\text{T}*\text{W}-0.00681103*{\text{W}}^{2} \end{array}\;(6)$$

With the aim of obtaining a statistically better model, Dixon test and Shapiro-Wilk test were performed to search for any outlier in the runs of the experimental design. According to the Dixon test, there are two outliers in the responses of yields of FAAs from viscera and total protein (runs 6 and 13), while the Shapiro-Wilk test indicates that the only response with a normal distribution is the degree of hydrolysis ( *p*-value of 0.888). Therefore, a model without the two outliers was obtained. Also, a reduced model was obtained eliminating the non-significant factors. The results were compared with the original model, both reduced and not ( Table 4 and Table 5). It can be observed that all results are similar except for the reduced model without outliers, which gets lower coefficients of determination. In all the cases, the *p* value of the Lack of Fit was above 0.05 for the degree of hydrolysis, which means that there is a good fit of the model for all cases, however, the *p*-value indicated that there was not a good agreement between the predicted and observed values. Therefore, the degree of hydrolysis was excluded from the optimization in the reduced models. The optimum parameters indicated in Table 5, except for the reduced model without outliers, were pH 8, temperature of 40 °C, and a content of added water less than 8%. In the reduced model without outliers, the temperature was found to be a non-significant factor, and two maximum responses were observed, one at pH 8 – 0 % of added water and another one at pH 6 – 50% of added water. Given the fact that there were no significant differences in the predicted values from the different models obtained, the parameters chosen for the validation were those of the original one.

**Table 4.** Comparison of the R ^2^ and Lack of Fit of the different models obtained with Statgraphics. P corresponds to pH, T corresponds to temperature and W corresponds to % of water added.

	Degree of hydrolysis			Yield of FAAs from viscera			Yield of FAAs from total protein		
	R ^2^	*p* value of the Lack of Fit	Significant coefficients	R ^2^	*p* value of the Lack of Fit	Significant coefficients	R ^2^	*p* value of the Lack of Fit	Significant coefficients
**Original** ** model**	79.7	0.4603	None	78.4	0.0046	W, P*P, P*W, T*W	83.7	0.022	P*W, W, W*W
**Reduced ** **original** ** model**	79.69	0.4603	P, P ^2^, P*T	74.84	0.0051	P P ^2^, P*T	81.06	0.004	W, P*W
**Model** ** without ** **outliers**	80.8	0.4037	None	81.6	0.0168	P ^2^, P*W, T*W, P, T	76.6	0.0647	P*W
**Model ** **without ** **outliers** ** reduced**	41.0	0.9983	None	65.26	0.0653	W, P, P*W	61.47	0.0590	W, P, P*W

**Table 5.** Comparison of the optimum parameters and the theoretical optimum responses obtained with Statgraphics.

	Optimum parameters			Predicted optimum values		
	pH	Temperature (°C)	Content of added water (%)	Degree of hydrolysis	Yield of FAAs from viscera	Yield of FAAs from total protein
**Original model**	8	40	6.85	68.8 ± 8.2	5.0 ± 1.0	62.8 ± 19.6
**Original model** ** reduced**	8	40	7.32	69.7 ± 6.7	4.9 ± 1.2	59.7 ± 9.5
**Model without** ** outliers**	8	42	0.01	67.5 ± 10.4	4.9 ± 1.1	58.3 ± 13.0
**Model without ** **outliers reduced**	8	50	0	64.1 ± 8.8	4.7 ± 0.7	57.8 ± 7.3

### Validation of the optimum conditions

**Figure 3.**
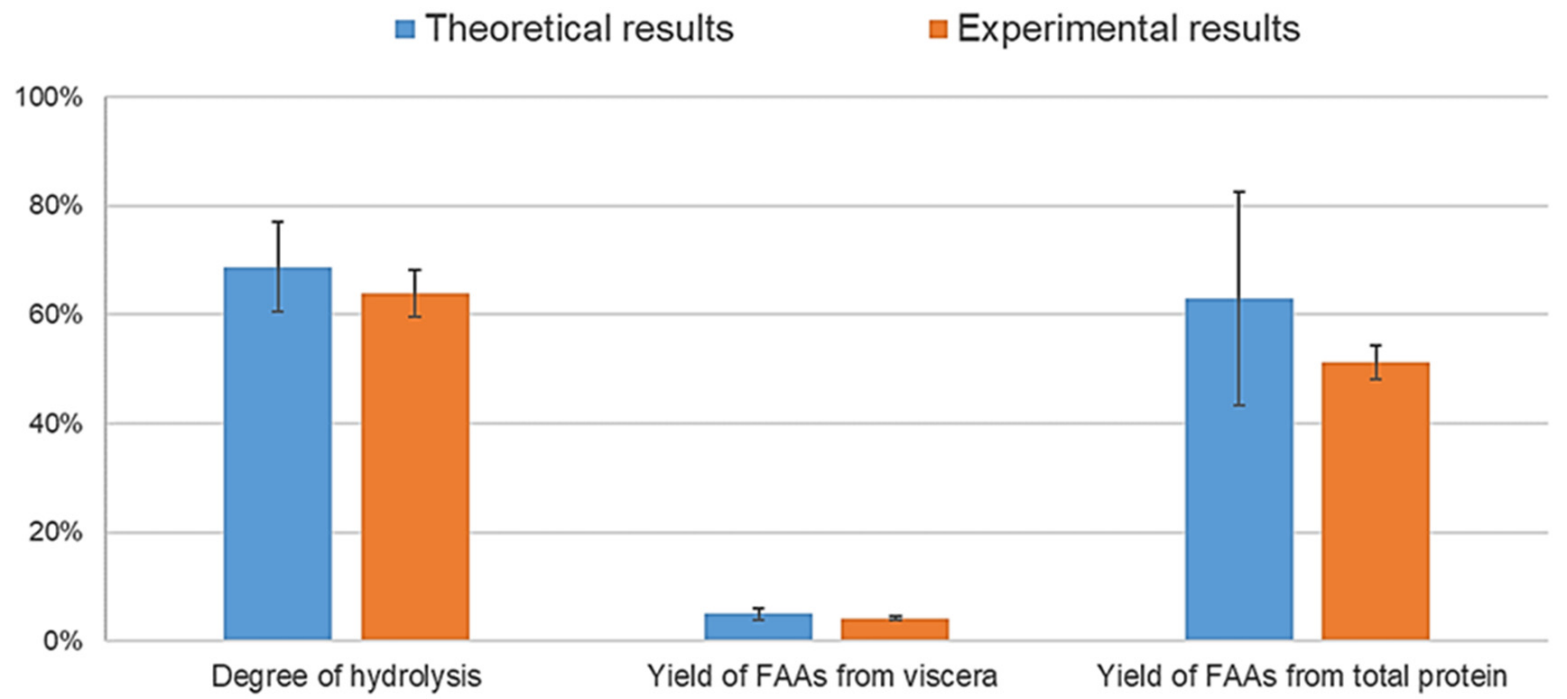
Comparison between theoretical and experimental results based on the optimum conditions (p>0.05, statistically comparable results).

To confirm the validity of the theoretical results provided by the Statgraphics software, three hydrolysis were carried out under the optimal conditions (pH 8, temperature of 40 °C, and content of water in the sample of 6.85%). The composition of the optimal hydrolysate is shown in Table 5, and a comparison between the theoretical and experimental results is shown in Figure 3. The graphs show that both results were not significantly different ( *p* > 0.05) according to the t Student’s t-test (confidence level of 95%) for the three variables, although the standard deviation of the theoretical results was high. This might be due to the high fat content in the raw material, which makes it difficult to obtain homogeneous samples and interferes with the enzymes in the hydrolysis process, as previously mentioned.

The profile of free amino acids in the dry matter was also determined ( Table 6), and glutamic acid (Glu) was the most abundant amino acid. This amino acid plays an important role in the biosynthesis of proline and other nitrogen-containing compounds, in addition to having a positive effect on photosynthetic activity and major production of fruits in plants ( Lv *et al.*, 2009; Serna-Rodríguez *et al.*, 2011). Therefore, the autolysis hydrolysate can be used as an intermediary product to produce amino acid-based biostimulants. The synthesis of amino acids requires high energy consumption in plants; therefore, foliar application is a common practice ( Shahrajabian *et al.*, 2022). According to Spanish legislation (RD 506/2013), a minimum of 6% of free amino acids on dry matter basis is required for the composition of a biostimulant or a product labeled as amino acids. In this case, the percentage of free amino acids in dry matter was 35.4 ± 1.8%. According to Shahrajabian *et al.* (2022), the amino acids that are usually used to formulate biostimulant products are tyrosine, methionine, and lysine, which are present at lower concentrations in the hydrolysate.

**Table 6.** Profile of free amino acids in dry matter (%) of the optimum protein hydrolysate, n=3.

	Amino acid (%) d.m.
**Glutamic acid**	6.7 ± 0.4
**Alanine**	3.3 ± 0.2
**Valine**	2.7 ± 0.1
**Glycine**	2.4 ± 0.1
**Serine**	2.4 ± 0.1
**Leucine**	2.4 ± 0.1
**Aspartic acid**	2.3 ± 0.1
**Threonine**	2.1 ± 0.1
**Asparagine**	1.8 ± 0.1
**Isoleucine**	1.8 ± 0.1
**Proline**	1.5 ± 0.3
**Lysine**	1.4 ± 0.1
**Methionine**	0.8 ± 0.02
**Arginine**	0.8 ± 0.1
**Histidine**	0.7 ± 0.03
**Phenylalanine**	0.7 ± 0.01
**Glutamine**	0.5 ± 0.03
**Tyrosine**	0.4 ± 0.03
**Hydroxyproline**	0.3 ± 0.02
**Tryptophan**	0.2 ± 0.06
**Cysteine**	0.2 ± 0.06
**TOTAL**	**35.4 ± 1.8**

### Kinetics study of the autolysis

A kinetics study of seven hours was carried out with the aim of reducing the hydrolysis time, thereby mitigating the associated economic costs. Samples were collected at various time points throughout the study (0, 1, 3, 5, and 7 h).

**Figure 4.**
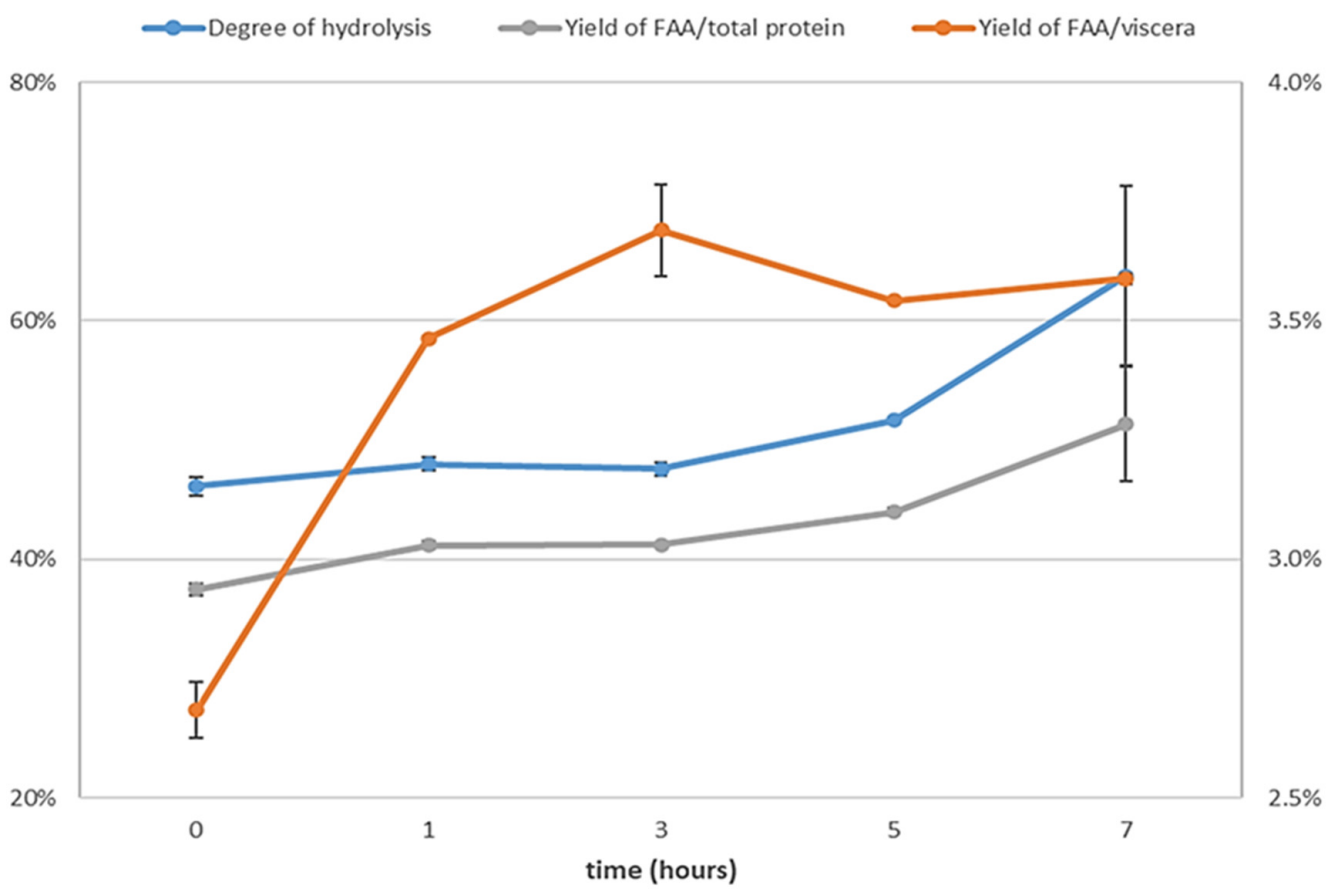
Evolution of autolysis through time. Degree of hydrolysis (left Y axis), yield of free amino acids from total protein (left Y axis) and yield of free amino acids from viscera (right Y axis), n=3.

**Figure 5.**
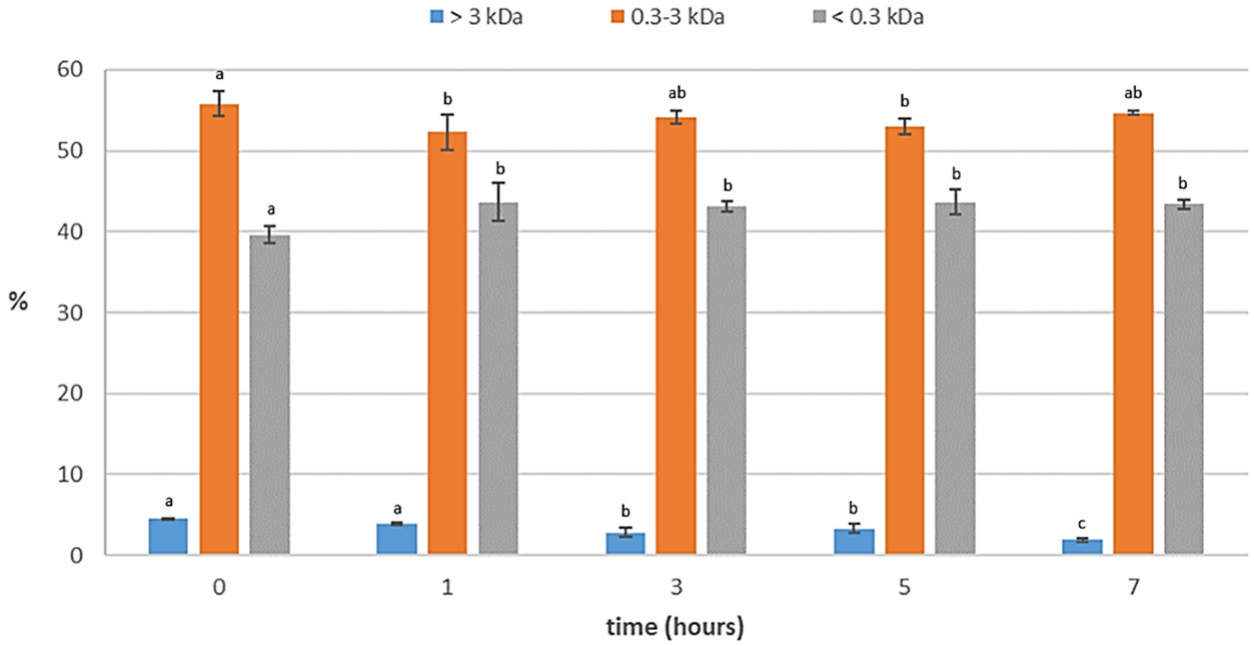
Profile of molecular weight distribution (%) of protein of the kinetics study of the autolysis (n=3). Different small superscript letters indicate significant differences (p<0.05).

In Figure 4, a high increase in free amino acids from viscera can be observed in the first hour, followed by a relatively modest rise, thereafter, maintaining stability over time. This observation is in accordance with the findings of Nikoo *et al.* (2021), who reported that the highest rate of peptide bond cleavage occurred within the first hour of autolysis and that the concentration of dipeptides and tripeptides increased notably in the first 3 h of autolysis. It can also be observed that the degree of hydrolysis and the yield of free amino acids from total protein increased during the last two hours, and at the same time, peptides larger than 3 kDa were cleaved ( Figure 5). This suggests that while the solubilization of protein ceased after three hours, hydrolysis of the protein present in the liquid phase persisted. Consequently, a complete autolysis process required 7 h to achieve optimal effectiveness. The activity of the endogenous enzymes (mainly trypsin) seemed to maintain their activity, despite a long autolysis time at pH 8 and 40 °C. In fact, according to Andevari *et al*. (2019), extracted proteases from rainbow trout viscera are highly stable at temperatures below 55 °C, retaining more than 87% of its activity after 60 minutes of heating at 40 °C and higher temperatures can provoke a loss of activity of almost 70% during the first hour.

### Comparison between autolysis, acid autolysis and enzymatic hydrolysis

The results obtained from the optimized autolysis were compared with the acid autolysis (7-day silage) and enzymatic hydrolysis (commercial enzyme Alcalase at 1% dose, Protana Prime at 1% dose, and endogenous enzymes for 7 h) carried out in previous experiments by Domínguez *et al.* (2024) ( Table 7). Enzymatic hydrolysis has emerged as the most efficient method, with the highest yield for all variables. Even if the degree of hydrolysis was achieved and the resulting concentration of FAA in the hydrolysate was lower, autolysis was comparable to silage in terms of the yield of FAAs from total protein present in viscera due to a higher yield of protein solubilized in the liquid fraction. Autolysis results suggested that more time might be required to achieve results comparable to those of silage. However, even when optimized, the efficiency of autolysis was far from the results of enzymatic hydrolysis and silage, as evidenced by the molecular weight distribution.

**Table 7.** Composition, profile of free amino acids (% of the total FAAs) and molecular weight distribution of the optimized autolysis, a 7-day acid autolysis, and an enzymatic hydrolysis, n=3. Different small superscript letters indicate significant differences (p<0.05).

	Optimized autolysis	ALC+PP+ENDO, 7h, 1%	7-day acid autolysis
**Dry matter**	16.8 ± 1.5 ^a^	8.6 ± 0.2 ^b^	19.9 ± 0.4 ^c^
**Protein d.m.**	55.7 ± 0.5 ^a^	63.9 ± 0.2 ^b^	73.8 ± 0.08 ^c^
**Ash d.m.**	11.9 ± 0.1 ^a^	7.1 ± 0.03 ^b^	5.2 ± 0.02 ^c^
**FAA d.m.**	35.4 ± 1.8 ^a^	50.3 ± 3.7 ^b^	49.2 ± 1.2 ^b^
**Glutamic acid**	19.0 ± 0.3 ^a^	12.9 ± 0.2 ^b^	10.0 ± 0.05 ^c^
**Alanine**	9.4 ± 0.1 ^a^	7.5 ± 0.1 ^b^	6.6 ± 0.04 ^c^
**Valine**	7.7 ± 0.1 ^a^	7.1 ± 0.1 ^b^	5.8 ± 0.01 ^c^
**Leucine**	6.8 ± 0.2 ^a^	9.5 ± 0.1 ^b^	9.2 ± 0.2 ^b^
**Glycine**	6.8 ± 0.1 ^a^	5.4 ± 0.03 ^b^	4.4 ± 0.2 ^c^
**Serine**	6.8 ± 0.1 ^a^	6.2 ± 0.05 ^b^	5.5 ± 0.1 ^c^
**Threonine**	6.0 ± 0.1 ^a^	5.4 ± 0.1 ^b^	4.9 ± 0.1 ^c^
**Aspartic acid**	5.1 ± 0.1 ^a^	4.5 ± 0.1 ^b^	4.5 ± 0.09 ^b^
**Asparagine**	5.1 ± 0.1 ^a^	4.3 ± 0.04 ^b^	3.4 ± 0.005 ^c^
**Isoleucine**	5.0 ± 0.1 ^a^	5.9 ± ± 0.1 ^b^	5.5 ± 0.04 ^c^
**Proline**	4.3 ± 0.8 ^a^	3.9 ± 0.2 ^a^	4.3 ± 0.5 ^a^
**Lysine**	4.0 ± 0.1 ^a^	4.9 ± 0.2 ^b^	8.8 ± 0.4 ^c^
**Methionine**	2.4 ± 0.1 ^a^	3.2 ± 0.03 ^a^	3.1 ± 0.1 ^a^
**Arginine**	2.2 ± 0.3 ^a^	4.8 ± 0.2 ^b^	7.5 ± 0.2 ^c^
**Histidine**	1.9 ± 0.1 ^a^	2.3 ± 0.04 ^b^	2.0 ± 0.001 ^a^
**Phenylalanine**	1.9 ± 0.1 ^a^	4.2 ± 0.1 ^b^	4.6 ± 0.1 ^c^
**Glutamine**	1.5 ± 0.1 ^a^	1.6 ± 0.1 ^a^	3.4 ± 0.03 ^b^
**Tyrosine**	1.2 ± 0.1 ^a^	3.1 ± 0.2 ^b^	3.3 ± 0.1 ^b^
**Hydroxyproline**	0.8 ± 0.1 ^a^	1.4 ± 0.3 ^a^	0.9 ± 0.5 ^a^
**Tryptophan**	0.4 ± 0.3 ^a^	1.9 ± 0.4 ^b^	1.7 ± 0.2 ^b^
**Cysteine**	0.2 ± 0.03 ^a^	0.4 ± 0.2 ^a^	0.4 ± 0.0001 ^a^
**Degree of hydrolysis (FAA/** **protein)**	63.8 ± 4.3 ^a^	83.8 ± 2.6 ^b^	75.8 ± 0.4 ^c^
**Yield of FAAs from viscera**	4.2 ± 0.3 ^a^	5.9 ± 0.1 ^b^	3.2 ± 0.002 ^c^
**Yield of FAAs from total** **protein in viscera**	51.2 ± 3.2 ^a^	71.3 ± 3.8 ^b^	52.5 ± 0.01 ^a^
**<0.3 kDa**	43.4 ± 0.6 ^a^	64.7 ± 0.5 ^b^	72.1 ± 0.2 ^c^
**0.3-1 kDa**	36.9 ± 0.02 ^a^	28.1 ± 0.1 ^b^	18.3 ± 0.1 ^c^
**1-3 kDa**	17.8 ± 0.2 ^a^	7.2 ± 0.4 ^b^	9.6 ± 0.2 ^c^
**3-6 kDa**	1.7 ± 0.1	< LOQ *	< LOQ *
**6-10 kDa**	0.2 ± 0.07	< LOQ *	< LOQ *

*Below limit of quantification

According to ANOVA, autolysis was significantly different for all parameters ( *p* < 0.05), except for proline (Pro), methionine (Met), histidine (His), glutamine (Gln), hydroxyproline (OHPro), and cysteine (Cys).

Regarding autolysis and acid autolysis, the pH values were completely different between the two cases (8 and 4, respectively). This means that the endogenous enzymes that cleaved the proteins were not the same. In the case of autolysis, serine proteases (trypsin and chymotrypsin) were the main acting enzymes, whereas acid autolysis acid proteases, such as pepsin, were the main involved enzymes.

In terms of environmental aspects, autolysis utilized less water compared to enzymatic hydrolysis (6.85% vs. 50%). However, autolysis required a larger quantity of concentrated sodium hydroxide to attain the desired pH of 8, whereas silage required formic acid to reach pH 4, and enzymatic hydrolysis only required a slight adjustment of pH to 7.

From an economic standpoint, both autolysis and silage present advantages over enzymatic hydrolysis because they do not require the use of commercial enzymes. Moreover, the heating of the sample during enzymatic hydrolysis and the higher amount of water to be evaporated to obtain the concentrated final product make the process the most expensive method.

In this context, the optimized autolysis method is a cost-effective approach to obtain an amino acid-rich hydrolysate within a relatively short timeframe, without the need for commercial enzymes. In addition, the low quantity of water utilized in autolysis contributes to its economic feasibility, making it an attractive option for producing valuable hydrolysates.

## Conclusions

In conclusion, response surface methodology was applied to the autolysis of rainbow trout viscera, and its optimization and validation were successful giving optimal conditions at pH 8, a temperature of 40 °C and a content of added water of 6.85%. Autolysis was efficient enough to obtain a protein hydrolysate in a shorter time than silage. In addition, the main differences between them were the pH and the endogenous enzymes that hydrolyzed the protein. Regarding enzymatic hydrolysis, the necessity of using commercial enzymes and higher water content may make it economically and environmentally disadvantageous comparing with the autolysis, despite obtaining higher yields. The autolysis conditions became more energy efficient and with reduced water use than enzymatic hydrolysis. However, complete comparative economic and life cycle assessments are required to confirm this. The free amino acid-rich hydrolysate obtained from the optimized autolysis could be used as an intermediate product for the formulation of biostimulants for plants, given its chemical composition. However, efficiency may still be subject to improvement to achieve higher yields of free amino acids, and the biostimulant effect of the resulting products should be verified in agronomic trials.

## Data Availability

Zenodo-Supplementary material to optimization of the autolysis of rainbow trout viscera for amino acid release using response surface methodology. SEA2LAND – Producing advanced bio-based fertilizers from fisheries wastes. DOI 10.5281/zenodo.11047418 Bald *et al.* (2024) Supplementary material to optimization of the autolysis of rainbow trout viscera for amino acid release using response surface methodology. This project contains the following underlying data related to this publication: Supplementary material Dominguez *et al.*, 2024 (2).xlsx Data are available under the terms of the Creative Commons Attribution 4.0 International license (CC-BY 4.0).”

## Software availability statement

The software used in this article, Statgraphics Centurion XVI, is a proprietary software. The statistical analysis performed in this article can be also performed using R. R is a free software environment for statistical computing and graphics https://www.r-project.org/

The response surface methodology (rsm) package is available from the Comprehensive R Archive Network at https://search.r-project.org/CRAN/refmans/rsm/html/rsm-package.html

Package rsm, was designed to provide R support for standard response-surface methods. Functions are provided to generate central composite and Box-Behnken designs as described by Lenth (2009).
