# Supplementary material for: Optimization of the autolysis of rainbow trout viscera for amino acid release using response surface methodology
Source: Open Res Eur. 2024 Nov 14;4:141. Originally published 2024 Jul 9. [Version 2] doi: 10.12688/openreseurope.17646.2 (PMC11587235; doi:10.12688/openreseurope.17646.2)
Supplement: Peer review history

# Reviewer report: Reviewer response for version 2

**DOI:** 10.21956/openreseurope.20468.r46274

**Published:** 2024-11-25

**Authors:** Sharon Nunal, Leonilo Jr. Endoma

**Affiliations:** 1 University of the Philippines Visayas, Miagao, Western Visayas, Philippines; 2 College of Fisheries and Ocean Sciences, University of the Philippines Visayas, Miagao, Western Visayas, Philippines

My comments and suggestions have been sufficiently addressed. I am recommending acceptance and indexing of the manuscript.

Is the study design appropriate and does the work have academic merit?

Yes

Is the work clearly and accurately presented and does it cite the current literature?

Partly

If applicable, is the statistical analysis and its interpretation appropriate?

Yes

Are all the source data underlying the results available to ensure full reproducibility?

Partly

Are the conclusions drawn adequately supported by the results?

Partly

Are sufficient details of methods and analysis provided to allow replication by others?

Partly

Reviewer Expertise:

Fisheries Microbiology and Biochemistry

We confirm that we have read this submission and believe that we have an appropriate level of expertise to confirm that it is of an acceptable scientific standard.

# Reviewer report: Reviewer response for version 2

**DOI:** 10.21956/openreseurope.20468.r46273

**Published:** 2024-11-21

**Authors:** Ulfah Amalia

**Affiliations:** 1 Department of Fisheries Product Technology,, Universitas Diponegoro, Semarang, Indonesia

I am writing to confirm my approval of the revised version of the manuscript titled, *"Optimization of the autolysis of rainbow trout viscera for amino acid release using response surface methodology"* for acceptance.

 After reviewing the revisions made in response to my previous comments, I am satisfied that the authors have adequately addressed the points raised and enhanced their study's clarity and rigor. The additional data and improved statistical analyses provide a more robust presentation of their findings, and the adjustments to the conclusions effectively align with the study’s objectives.

 Therefore, I am pleased to endorse the indexing of this revised article. Thank you for the opportunity to review this work.

Is the study design appropriate and does the work have academic merit?

Yes

Is the work clearly and accurately presented and does it cite the current literature?

Yes

If applicable, is the statistical analysis and its interpretation appropriate?

Partly

Are all the source data underlying the results available to ensure full reproducibility?

Yes

Are the conclusions drawn adequately supported by the results?

Yes

Are sufficient details of methods and analysis provided to allow replication by others?

Partly

Reviewer Expertise:

food engineering, protein biochemistry, seafood allergology

I confirm that I have read this submission and believe that I have an appropriate level of expertise to confirm that it is of an acceptable scientific standard.

# Reviewer report: Reviewer response for version 2

**DOI:** 10.21956/openreseurope.20468.r46272

**Published:** 2024-11-15

**Authors:** Bui Xuan Dong

**Affiliations:** 1 The University of Danang, University of Science and Technology, Danang, Vietnam

Dear Authors and Editorial Team,

 I am writing to confirm my approval of the revised version of the manuscript titled, *"Optimization of the autolysis of rainbow trout viscera for amino acid release using response surface methodology"* for publication.

 After reviewing the revisions made in response to my previous comments, I am satisfied that the authors have adequately addressed the points raised and have enhanced the clarity and rigor of their study. The additional data and improved statistical analyses provide a more robust presentation of their findings, and the adjustments to the conclusions effectively align with the study’s objectives.

 Therefore, I am pleased to endorse the indexing of this revised article. Thank you for the opportunity to review this work.

 Also, please email me a certificate confirming my peer review of this article at xdbui@dut.udn.vn, as I need this certificate for my professional work at the University.

 Best regards

Is the study design appropriate and does the work have academic merit?

Yes

Is the work clearly and accurately presented and does it cite the current literature?

Yes

If applicable, is the statistical analysis and its interpretation appropriate?

Yes

Are all the source data underlying the results available to ensure full reproducibility?

Yes

Are the conclusions drawn adequately supported by the results?

Yes

Are sufficient details of methods and analysis provided to allow replication by others?

Yes

Reviewer Expertise:

My ORCID: https://orcid.org/0000-0003-0039-7215. My research focuses on Food Biotechnology, encompassing: 1) The application of enzymatic processes in food; 2) The application of biochemical processes and fermentation in food; 3) The application of biochemical and microbial processes (including microalgae and bacteria) in managing food waste for novel food production and sustainable energy (such as biogas and biodiesel)

I confirm that I have read this submission and believe that I have an appropriate level of expertise to confirm that it is of an acceptable scientific standard.

## References

[1] : Optimization of production parameters of fish protein hydrolysate from Sarda Orientalis black muscle (by-product) using protease enzyme. *Clean Technologies and Environmental Policy* .2021;23(1) : 10.1007/s10098-020-01867-2 31-40 10.1007/s10098-020-01867-2

# Reviewer report: Reviewer response for version 1

**DOI:** 10.21956/openreseurope.19068.r42430

**Published:** 2024-08-07

**Authors:** Bui Xuan Dong

**Affiliations:** 1 The University of Danang, University of Science and Technology, Danang, Vietnam

**General comments:**

 This paper uses a novel and informative strategy. One of the areas of importance of enzyme technology is innovation production from rainbow trout viscera using enzyme technology (autohydrolysis). Autohydrolysis is a cheap and economical method for obtaining FAAs. The authors conducted, the optimal conditions for achieving the highest degree of hydrolysis and yield of free amino acids (FAAs) per 100 g of viscera and per total The optimal conditions for achieving the highest degree of hydrolysis and yield of free amino acids (FAAs) per 100 g of viscera and per total.

  The study’s hypothesis and concept in protein autolysis and enzyme technology (for example, the optimal conditions for achieving the highest degree of hydrolysis, and yield of free amino acids (FAAs), and also rainbow trout viscera and their environmental impact) are interesting. The aim of the work is very clear. Here I would like to offer some comments as follows:

**Specific comments:**

 1. This response surface methodology (RSM) is not reasonable because the response surfaces of Fig. 2 B and Fig. 2 C do not converge;

 2. Recommendation: Add more samples for Experimental planning based on Box–Behnken virtual variable design (specifically, yield of FAAs from viscera and yield of FAAs from total protein);

Is the study design appropriate and does the work have academic merit?

Yes

Is the work clearly and accurately presented and does it cite the current literature?

Yes

If applicable, is the statistical analysis and its interpretation appropriate?

Yes

Are all the source data underlying the results available to ensure full reproducibility?

Yes

Are the conclusions drawn adequately supported by the results?

Yes

Are sufficient details of methods and analysis provided to allow replication by others?

Yes

Reviewer Expertise:

My ORCID: https://orcid.org/0000-0003-0039-7215. My research focuses on Food Biotechnology, encompassing: 1) The application of enzymatic processes in food; 2) The application of biochemical processes and fermentation in food; 3) The application of biochemical and microbial processes (including microalgae and bacteria) in managing food waste for novel food production and sustainable energy (such as biogas and biodiesel)

I confirm that I have read this submission and believe that I have an appropriate level of expertise to confirm that it is of an acceptable scientific standard, however I have significant reservations, as outlined above.

# Response

**Published:** 2024-11-05

**Authors:** Carlos Bald

**Affiliations:** Food Research, AZTI Foundation, Derio, Bizkaia, Spain

Thank you very much for taking the time to review this manuscript. Please find the detailed responses below and the corresponding revisions/corrections highlighted in track changes mode in the re-submitted files.

 1. This response surface methodology (RSM) is not reasonable because the response surfaces of Fig. 2 B and Fig. 2 C do not converge.

 Author response: We do not understand well what you mean. In fig 2b and 2c, we established the maximum pH at 8 because, as we mention, in a previous experiment pH>8 caused a change in the consistency of the sample making the hydrolysis impossible. Anyway, we can say that the response FAA/viscera weight is a parameter which depends on the variability in raw material composition, while FAA/total protein is independent of it but is affected by a variability in the fat content, because it affects enzymes performance. The lack of homogeneity in the sample composition may explain why both responses show slight differences. As it can be seen, the effect of the temperature was found to be non-significant.

 2. Recommendation: Add more samples for Experimental planning based on Box–Behnken virtual variable design (specifically, yield of FAAs from viscera and yield of FAAs from total protein)

 Author response: This is an overall good suggestion; however, we think that it is not necessary since, as explained now in the article, due to the high variability in the sample composition this would not guarantee obtaining better fitting of the model. Thus, we have obtained a model by eliminating two outliers and the optimal parameters are similar, and the predicted optimal values do not differ significantly from the original and reduced model.

# Reviewer report: Reviewer response for version 1

**DOI:** 10.21956/openreseurope.19068.r42116

**Published:** 2024-08-01

**Authors:** Sharon Nunal, Leonilo Jr. Endoma

**Affiliations:** 1 University of the Philippines Visayas, Miagao, Western Visayas, Philippines; 2 College of Fisheries and Ocean Sciences, University of the Philippines Visayas, Miagao, Western Visayas, Philippines

The study has a good potential of contributing new knowledge and information that could be beneficial to waste management efforts and to various fish processing industries. I have minor comments as follows: 

 1. Change some old references (eg. 1994) to recent ones. 

 2. Indicate the reference of some methods especially those that are adapted (eg. measurement of the degree of hydrolysis)

 3. Clarify the difference between yield of FAA from viscera and from total protein. What is the importance of measuring both?

 4. Why is hydrolysis time not considered as a factor for optimization? If it was done, then there was no need for the kinetics study. 

 5. I have reservations regarding the generated models because the coefficients of determination are quite low. This implies that the models cannot be used to predict responses. Authors should consider model reduction or addition to improve the R2. 

 Here are the additional comments from my co-reviewer: 

 1. Are the results in this study presented as Mean±Standard Deviation? (n=3?). Statgraphics Centurion XVI software (16.2.04 version, Statgraphics Technologies Inc., Virginia, USA) was used to perform the experimental design and statistical analysis. RSM ANOVA was used to determine significant terms included in the model (at p<0.05) while having a Lack of Fit (LoF) p-value >0.05 and an R ^2^ at least 0.9. For the validation of the optimum conditions, results were analyzed with analysis of variance (ANOVA) and Student’s t-test, and factors were considered significant at *p*< 0.05. 

 2. Shouldn't it be Results **and Discussion?**

 3. Table and Figure description is incomplete. The equations of the adjusted model were based on coded or actual values?

 4. Kindly check/ measure the enzyme activity. Indicate the [crude proteolytic] enzyme activity. "The activity of the endogenous enzymes (mainly trypsin) seemed to maintain their activity (add values), despite a long autolysis time at pH 8 and 40°C.”

 5. Explain further why the DH, yield of FAA from viscera and from total protein were low at 50°C, pH 6.0, and with no added water (0%)? Conversely, what causes some conditions to have high DH, and high yield of FAA from viscera and from total protein. 

 6. Kindly check the Figure Labels in Figure 2. Although there are numerous graphs presented, I would like to check whether the labels are correctly placed.

 7. In Figure 2, kindly check if the water content, pH and temperature are varied and  not  fixed at a certain value.

 8.  In the conclusion, what is then the optimum condition for autolysis of rainbow trout viscera for amino acid release?’ The study should have highlighted the practical application of using autolysis and manipulating the conditions to increase free amino acid release (from viscera or from protein) and DH.

Is the study design appropriate and does the work have academic merit?

Yes

Is the work clearly and accurately presented and does it cite the current literature?

Partly

If applicable, is the statistical analysis and its interpretation appropriate?

Yes

Are all the source data underlying the results available to ensure full reproducibility?

Partly

Are the conclusions drawn adequately supported by the results?

Partly

Are sufficient details of methods and analysis provided to allow replication by others?

Partly

Reviewer Expertise:

Fisheries Microbiology and Biochemistry

We confirm that we have read this submission and believe that we have an appropriate level of expertise to confirm that it is of an acceptable scientific standard, however we have significant reservations, as outlined above.

# Response

**Published:** 2024-11-05

**Authors:** Carlos Bald

**Affiliations:** Food Research, AZTI Foundation, Derio, Bizkaia, Spain

Thank you very much for taking the time to review this manuscript. Please find the detailed responses below and the corresponding revisions/corrections highlighted in track changes mode in the re-submitted files.

**1. Change some old references (eg. 1994) to recent ones.** 

 Author response: There are two references from this year. One of them has been removed as it was a secondary reference. The other one (Fisheries Processing) has been kept because of its relevance in the literature.

**2. Indicate the reference of some methods especially those that are adapted (eg. measurement of the degree of hydrolysis).**

 Author response: In this work, the degree of hydrolysis was not measured but calculated. The calculation of the degree of hydrolysis and the two other chosen responses of the RSM have been explained through their respective equations in the Section of Results and Discussion.

**3. Clarify the difference between yield of FAA from viscera and from total protein. What is the importance of measuring both?**

 Author response: There are two ways of measuring the efficiency of the autolysis process. The yield of FAAs from viscera explains how much FAA content can be extracted from the viscera sample, and it shows the overall yield of the autolysis process directly related to the quantity of available raw material. We have chosen to display it due it is a parameter used at industrial level. FAA/viscera weight is affected by the viscera proximal composition and by its variability. The yield of FAAs from total protein explains how much FAAs can be extracted from the protein contained in the sample, considering how much protein is solubilised and from this, how much of that protein is hydrolysed independently of the viscera composition. The variability in the viscera composition will also affect this parameter because the oil hinders the action of the enzymes.

**4. Why is hydrolysis time not considered as a factor for optimization? If it was done, then there was no need for the kinetics study.** 

 Author response: For economy reasons and because we wanted to compare the results with the previous experiments done with commercial enzymes in which we fixed the hydrolysis time in 7 hours. While pH, temperature and added water are strictly crucial factors that determine the efficiency of the autolysis, time can be simply extended or shorten using the optimum conditions of the beforementioned factors. Therefore, the kinetics study carried out after the optimization was used to clarify if, with the optimized conditions, the autolysis could be shortened in time.  

**5. I have reservations regarding the generated models because the coefficients of determination are quite low. This implies that the models cannot be used to predict responses. Authors should consider model reduction or addition to improve the R2.** 

  Author response: We agree that the R2 coefficients are not very high, but we have explained in the article that it was probably due to the complexity of the sample and the difficulty of the homogenous sample preparation.  

**Here are the additional comments from my co-reviewer:** 

**1. Are the results in this study presented as Mean±Standard Deviation? (n=3?). ** n=3  added. **Statgraphics Centurion XVI software (16.2.04 version, Statgraphics Technologies Inc., Virginia, USA) was used to perform the experimental design and statistical analysis. RSM ANOVA was used to determine significant terms included in the model (at p<0.05) while having a Lack of Fit (LoF) p-value >0.05 and an R ^2^ at least 0.9. For the validation of the optimum conditions, results were analyzed with analysis of variance (ANOVA) and Student’s t-test, and factors were considered significant at *p*< 0.05.**

 Author response: We agree that R2 is slightly low and P value for R2 was not significant for the three responses. As for both, FAA/viscera ad FAA/ total protein, pH, and % water added were found affecting significantly the response, we decided to eliminate the temperature as factor in the equation. P value was then less than 0.05 except for the degree of hydrolysis R2 slightly decreased but adjusted R2 improved for all the responses. As mentioned, due the lack of homogeneity in the sample composition, as other authors report, better results in the R2 are not possible. A model without outliers and a reduced model were also considered to improve the model statistically, but when calculating the optimal values, R2 and Lack of Fit for the three responses, the values were similar to those of the full model. Therefore, the full model (the original) was considered for its use in the validation. A comment and two explanatory tables have been added to the article to explain this.

**2. Shouldn't it be Results and Discussion?**

 Author response: We agree, the title has been changed.

**3. Table and Figure description is incomplete. The equations of the adjusted model were based on coded or actual values?**

 Author response: They were actual values.  X1, X2 and X3 have been substituted with letters P, T and W to make the reading of the equations easier.

**4. Kindly check/ measure the enzyme activity. Indicate the [crude proteolytic] enzyme activity. "The activity of the endogenous enzymes (mainly trypsin) seemed to maintain their activity (add values), despite a long autolysis time at pH 8 and 40°C.”**

 Author response: This statement is based on the observation that the hydrolysis continued increasing steady during all the 7 hours of autolysis. As other authors mention, the extracted proteases from rainbow trout viscera are highly stable at temperatures below 55 °C, retaining more than 87% of its activity after 60 min of heating at 40 °C, which is our case. We have modified this sentence trying to clarify it. We do not consider necessary to measure the activity through the standard method using casein as a substrate because we are giving a measurement of the DH and the FAA extracted per 100 g of viscera protein.

**5. Explain further why the DH, yield of FAA from viscera and from total protein were low at 50°C, pH 6.0, and with no added water (0%)? Conversely, what causes some conditions to have high DH, and high yield of FAA from viscera and from total protein. ** Author response: Briefly commented in section Results and Discussion.

**6. Kindly check the Figure Labels in Figure 2. Although there are numerous graphs presented, I would like to check whether the labels are correctly placed.**

 Author response: Checked, everything is alright.

**7. In Figure 2, kindly check if the water content, pH and temperature are varied and not fixed at a certain value.**

 Author response: In all the graphs, there is one variable that is kept fixed at its central point and is displayed so in Fig. 2

**8.  In the conclusion, what is then the optimum condition for autolysis of rainbow trout viscera for amino acid release?’ The study should have highlighted the practical application of using autolysis and manipulating the conditions to increase free amino acid release (from viscera or from protein) and DH.**

 Author response: Optimum conditions are now explained in the Conclusions. The objective is further clarified in the introduction (last paragraph) **.**

# Reviewer report: Reviewer response for version 1

**DOI:** 10.21956/openreseurope.19068.r42428

**Published:** 2024-07-31

**Authors:** Ulfah Amalia

**Affiliations:** 1 Department of Fisheries Product Technology,, Universitas Diponegoro, Semarang, Indonesia

Overall, the manuscript gave clearly information due protein hydrolysates from fish viscera.

 I have several notes in this manuscript as below:

 1. In introduction part, specifically in paragraph 2, the author did not mention the amount of rainbow trout by-products or at least, mention  a product derivated from rainbow trout.

 2. Regarding my comment (partly) for method to quantify the yield of protein hydrolysis degree and yield of FAA did not explain in detail. Therefore the authors should complete the explanation.

 3. Based on Table 3, it is appeared that the lowest temperature (40 C) in the autolysis process results higher degree of protein hydrolysis compared to the other higher temperature (50 & 60 C) even in the 50 C there was a highest degree of protein hydrolysis (72.5%) with the amount of water added as same as in 40 C. What is the reason?

 4. The statement result in section of "Optimization of autolysis conditions": The optimum conditions according to the RSM were pH 8, a temperature of 40 °C, and a 6.85% water content in the sample, with the following predicted results: 68.8% degree of hydrolysis, yield of 5.0% FAAs from viscera, and yield of 62.8% FAAs from total protein.

 The question: Where the pH 4 and 6.85% water content came from? In the treatment, there were only three different treatments of pH (6, 7, & 8) and also three different treatment of water addition which are 0, 25, and 50%. 

 5. Profile of free amino acids as appeared in the Table 4 was continue with the explanation of the potential of amino acid in each. However, the discussion is limited only on plant application, while in the introduction, author mention several sectors such as application in nutraceuticals, animal feed, etc. Could author add the reference for those mentioned issues?

 6. In conclusion, it would be better if the author could clearly mention the fix (chosen) treatment based on the study for example in what pH, temperature and how much water should add in.

Is the study design appropriate and does the work have academic merit?

Yes

Is the work clearly and accurately presented and does it cite the current literature?

Yes

If applicable, is the statistical analysis and its interpretation appropriate?

Partly

Are all the source data underlying the results available to ensure full reproducibility?

Yes

Are the conclusions drawn adequately supported by the results?

Yes

Are sufficient details of methods and analysis provided to allow replication by others?

Partly

Reviewer Expertise:

food engineering, protein biochemistry, seafood allergology

I confirm that I have read this submission and believe that I have an appropriate level of expertise to confirm that it is of an acceptable scientific standard, however I have significant reservations, as outlined above.
